# Predictive Scores for Early Identification of Immune-Mediated Thrombotic Thrombocytopenic Purpura: Room for Improvement?

**DOI:** 10.1016/j.ekir.2022.09.016

**Published:** 2022-09-23

**Authors:** Adrien Joseph, Benoit Brilland, Laure Burguet, Martin Eloit, Nicolas Fage, Jean-François Augusto, Yahsou Delmas, Agnès Veyradier, Jean-Michel Halimi, Paul Coppo

**Affiliations:** 1 Service de Médecine intensive réanimation, Hôpital Saint Louis, AP-HP, Paris, France; 2 Centre de Référence des Microangiopathies Thrombotiques (CNR-MAT), AP-HP, Paris, France; 3 Service de Néphrologie-Dialyse-Transplantation, Université d’Angers, CHU Angers, Angers, France; 4 Service de Néphrologie, CHU de Bordeaux, Bordeaux, France; 5 Service d’Hématologie et Thérapie Cellulaire, CHRU de Tours, France; 6 Service d’Hématologie Biologique, Hôpital Lariboisière, AP-HP, Paris, France; 7 Service de Néphrologie-hypertension, Dialyses, Transplantation Rénale, Hôpitaux Bretonneau et Clocheville, Tours, France; 8 Service d’Hématologie, Hôpital Saint-Antoine, AP-HP, Paris, France

To the Editor:

Thrombotic microangiopathies (TMAs) have separate nosologic entities with distinct pathophysiology and treatment. However, clinical overlap and nonreducible time for specific biological assays such as *ADAMTS13* evaluation may preclude rapid diagnosis of TMA syndromes. To circumvent these limits, clinical scores on the basis of baseline features have been developed, with a focus on identification of patients with immune-mediated thrombotic thrombocytopenic purpura (iTTP). The most widely used, namely the French^1^ and the PLASMIC scores, have been recently included in the International Society on Thrombosis and Haemostasis (ISTH) guidelines.^2^

Diagnostic performances of these scores are concordant. Nevertheless, patients with an intermediate score (French score 1 or PLASMIC score 5) still represent a diagnostic issue, because their probability of iTTP remains undetermined. Therefore, strategies to improve the diagnostic performances of these scores are urgently needed. Recently, 2 additional factors were suggested to improve scores’ performances. First, 2 studies reported that proteinuria improved diagnostic performances of both French and PLASMIC scores.^3^^,^^4^ By pooling patients from these studies (*N* = 123) (excluding pregnancy-related TMA), we report that patients with a French score of 1 had a 20% positive predictive value for iTTP diagnosis, which increased to 41% when proteinuria was <1.2 g/g and decreased to 4% if this ratio was ≥1.2 g/g, (*P* = 0.011, χ^2^ test) (Table 1). In another study, the analysis of blood pressure (BP) for patients with TMA (*n* = 539) showed that BP at admission was lower in patients with iTTP.^5^ In this study, a French score of 1 had a 56% positive predictive value for iTTP, which increased to 63% if systolic BP was ≤150 mmHg and decreased to 36% with a BP >150 mmHg (*P* = 0.021, χ^2^ test) (Table 1). Of note, older (≥60 years old) patients also benefited from the addition of BP and proteinuria criteria (Supplementary Table S1).

**Table 1:** Probability of immune-mediated thrombotic thrombocytopenic purpura according to the French score before and after addition of systolic blood pressure or proteinuria criteria and diagnostic values of these criteria for patients with an intermediate score

Performances	Joseph *et al.*^5^ (*n* = 539 TMAs)				Fage *et al.*^3^ and Burguet *et al.*^4^ (*n* = 123 TMAs)			
	French score				French score			
	0	1		2	0	1		2
Proportion of iTTP (Severe *ADAMTS13* deficiency-proven iTTP/total TMAs by score)	15/139 11%	60/107 56%		282/293 96%	2/38 5%	8/41 20%		35/44 80%
		Systolic blood pressure				Proteinuria		
		> 150 mm Hg	≤ 150 mm Hg			≥ 1.2 g/g	< 1.2 g/g	
		10/28 36%	50/79 63%			1/24 4%	7/17 41%	
Sensibility		83%				88%		
Specificity		38%				70%		
Positive likelihood ratio		1.35				2.89		
Negative likelihood ratio		0.44				0.18		

iTTP, immune-mediated thrombotic thrombocytopenic purpura; TMAs, thrombotic microangiopathies.

At a time when targeted therapies are increasingly available, clinical scores coupled with *ADAMTS13* activity are becoming standard clinical practice.^2^ In this context, the algorithm of intermediate scores should be fine-tuned with the use of proteinuria and BP. Evaluation of the additive value of these 2 factors and external validation are needed before implementation in clinical practice.

## Supplementary Material

Supplementary File (PDF)

**Table S1.** Probability of immune-mediated thrombotic thrombocytopenic purpura according to the French score before and after addition of systolic blood pressure or proteinuria criteria and diagnostic values of these criteria for older patients (≥60-year-old) with an intermediate score.
