# Supplementary material for: Predictive Scores for Early Identification of Immune-Mediated Thrombotic Thrombocytopenic Purpura: Room for Improvement?
Source: Kidney Int Rep. 2022 Sep 23;7(11):2541–2. doi: 10.1016/j.ekir.2022.09.016 (PMC9751677; doi:10.1016/j.ekir.2022.09.016)
Supplement: Supplementary File (PDF) [file mmc1.pdf]

	***Joseph et al.* (n= 111 TMAs)**				***Fage et al. & Burguet et al.* (n= 52 TMAs)**			
	French score				French score			
	0	1		2	0	1		2
Proportion of iTTP (Severe ADAMTS13 deficiency-proven iTTP/total TMAs by score)	5/47 11%	9/29 31%		31/35 89%	0/9 0%	3/25 12%		15/18 83%
		Systolic blood pressure				Proteinuria		
		> 150 mmHg	≤ 150 mmHg			≥ 1.2 g/g	< 1.2 g/g	
		3/14 21%	6/15 40%			0/14 0%	3/11 27%	
Sensibility		67%				100%		
Specificity		55%				64%		
Positive likelihood ratio		1.48				2.75		
Negative likelihood ratio		0.61				0		

Supplementary Table S1: Probability of immune-mediated thrombotic thrombocytopenic purpura according to the French score before and after addition of systolic blood pressure or proteinuria criteria and diagnostic values of these criteria for older patients (≥60-year-old) with an intermediate score.
